# Epigenetic studies in children at risk of stunting and their parents in India, Indonesia and Senegal: a UKRI GCRF Action Against Stunting Hub protocol paper

**DOI:** 10.1136/bmjpo-2022-001770

**Published:** 2024-02-27

**Authors:** Anouschka S Ramsteijn, Magatte Ndiaye, Rajender Rao Kalashikam, Min Kyaw Htet, Dinesh Yadav DM, Little Flower Augustine, Nur L Zahra, Aicha Djigal, Dwi Yanti, Tiffany C Angelin, Mifa Nurfadilah, Manjula Gorre, Dantham Subrahamanyam, Sai Santhosh Vadakattu, Manne Munikumar, Graham W Horgan, Umi Fahmida, Babacar Faye, Bharati Kulkarni, Paul Haggarty

**Affiliations:** 1 Rowett Institute, University of Aberdeen, Aberdeen, UK; 2 Service de Parasitologie-Mycologie, Faculté de Médecine, Université Cheikh Anta Diop (UCAD), Dakar, Senegal; 3 ICMR-National Institute of Nutrition, Hyderabad, India; 4 South East Asian Ministers of Education Organization Regional Centre for Food and Nutrition (SEAMEO RECFON), East Jakarta, Indonesia; 5 Biomathematics and Statistics Scotland, Aberdeen, UK

**Keywords:** Genetics, Growth, Data Collection

## Abstract

**Introduction:**

In 2020, an estimated 150 million children under the age of 5 years were stunted. Stunting results from early-life adversity and it is associated with significant physical and cognitive deficit, lifelong socioeconomic disadvantage and reduced life expectancy. There is a need to understand the causes of stunting and its effects in order to develop strategies to avoid it and to mitigate the consequences once stunting has occurred. Epigenetics is an important mechanism through which early-life factors are thought to influence biological function, with long-term consequences. We describe a series of epigenetic studies designed to understand how early-life adversity results in stunting and to inform the development of practical tools such as predictive markers and therapeutic targets. This work is part of the UKRI GCRF Action Against Stunting Hub.

**Methods and analysis:**

The project—in India, Indonesia and Senegal—comprises an observational study of mothers, fathers, and offspring (n=500) spanning the first 1000 days of life, and an intervention study in each country. Epigenetic status (DNA methylation) is determined in saliva from babies collected within 1 month of birth and again at 18 months of age, and from mothers and fathers around the time of birth. Epigenome-wide analysis is carried out using the Illumina EPIC array, augmented by high-definition sequencing approaches. Statistical analysis is carried out at the level of candidate genes/regions, higher dimensional epigenetic states and epigenome-wide association. Data analysis focuses on the determinants of stunting, the effectiveness of interventions, population comparisons and the link between epigenetics and other thematic areas, which include anthropometry, microbiome, gut health, parasitology, cognition, nutrition, food hygiene and water sanitation, food systems and the home environment.

**Ethics and dissemination:**

This study has been approved by the relevant Ethics Committees in Indonesia, India and Senegal, and the UK. Research data will be published and posted in public repositories.

**WHAT IS ALREADY KNOWN ON THIS TOPIC**

- Child stunting is a significant global problem that has proved resistant to policy interventions.
- There is little understanding of how early-life adversity results in stunting, what can be done to avoid it and how outcomes can be improved.

**WHAT THIS STUDY ADDS**

- A deeper understanding of the biology of stunting and the role of epigenetics.
- A holistic understanding of stunting that considers the epigenetics of the family unit (children, mothers and fathers).

**HOW THIS STUDY MIGHT AFFECT RESEARCH, PRACTICE OR POLICY**

- This study will generate practical epigenetic tests of stunting risk, assess the epigenetic response to interventions, and identify epigenetic targets amenable to modification.

## Introduction

In 2020, almost 150 million children worldwide under 5 years of age suffered from stunting (defined as being more than 2 SD below the WHO height-for-age standards). The effect of the COVID-19 pandemic on stunting rates will not be fully understood for some years^1^ but it has been estimated that COVID-19-related disruptions in health and nutrition services will further increase the number of stunted children in low-income and middle-income countries (LMICs) by around 2.6 million by 2022.^2^ The physical and cognitive deficits linked to stunting, and the long-term health and socioeconomic consequences for the children affected, are both significant and potentially avoidable. The scale of the problem represents a major loss of human capital in precisely those countries that need it most.

In order to address the global problem of stunting there is a need to: (1) understand the causes of stunting in order to develop strategies to avoid it; (2) predict the risk of stunting in order to facilitate early intervention; and (3) identify children and populations affected by stunting that are able to respond to specific interventions. Not all children in LMICs who experience adversity in early life become stunted and it is important to understand how they escape the physical consequence of stunting and whether they exhibit other biological adaptations. This paper describes a series of epigenetic studies in populations subject to significant rates of stunting in order to address these issues (box 1) in a way that encompasses the full complexity of the causes and consequences of stunting across multiple domains and disciplines (see for example Assessment of the role of gut health in childhood stunting in a multisite, longitudinal study in India, Indonesia, and Senegal: a UKRI GCRF Action Against Stunting Hub protocol. *BMJ Paeds Open, Kadia et al (in press*); Anthropometric, biochemical, dietary, morbidity and wellbeing assessments in women and children in Indonesia, India and Senegal: a UKRI GCRF Action Against Stunting Hub protocol paper. *BMJ Paeds Open, Davies-Kershaw et al (in press*)).

Box 1 **Practical aims of the epigenetic work within the Action Against Stunting Hub**

**Predict:**

- Use epigenetic tests to identify children and pregnancies already on the pathway to stunting to prioritise for early intervention.

**Ameliorate or reverse:**

- Develop interventions based on epigenetic type characteristic of stunting.
- Use epigenetic type to predict the ability of the stunted child to respond to specific interventions.

**Avoid:**

- Develop strategies to avoid stunting before it has occurred based on antecedents and their effects on epigenetic type.
- Understand the role of epigenetics in children who are biologically resistant to stunting to inform strategies to avoid stunting in vulnerable children.

Epigenetics represents a linking mechanism which is both responsive to the environment and parental factors and which has the potential to explain the physical and mental manifestations of stunted development. Crucially, unlike genetics, epigenetic offers hope to the millions already stunted, or at risk of stunting, as many epigenetic states are potentially reversible. The focus of this study is on modifiable risk and epigenetics but there is also crosstalk between the genetic sequence and the epigenetic code. Genetic information produced during sequencing will be included in the statistical models where appropriate to identify or rule out genetic explanations for significant epigenetic results.

Epigenetics refers to the information in the genome over and above the purely genetic information contained in the DNA base sequence.^3^ The characteristics of epigenetics make it particularly suited to research problems such as stunting that span multiple domains. It is increasingly recognised as an important mechanism through which early-life factors can influence biological function with long-term consequences. Epigenetics represents the distillation and integration of the exposures and the biological response implicated in stunting. Epigenetic regulation is essential for neurogenesis, brain function and behaviour,^4–7^ it affects in-utero and postnatal growth,^8–10^ it is central to nutritional metabolism^9–14^ and immune function,^15^ it responds to a wide range of environmental exposures,^16–18^ and it determines longevity and the health trajectory across the life-course.^19–21^

Stunting often begins before birth and there is a recurrent intergenerational component whereby women who were themselves stunted in childhood are at greater risk of bearing stunted offspring.^22^ Epigenetics is the primary candidate mechanism to explain such intergenerational phenomena^6 9 10 23^ and it has specifically been proposed as the explanation for the apparent heritability of stunting.^22^

## Methods and analysis

The research is carried out in three countries (India, Indonesia and Senegal). It comprises a large observational study of mothers, fathers, and offspring, spanning the first 1000 days of life, and a series of intervention studies. In addition to the scientific aims, the project is designed to train scientists from LMICs in cutting-edge methodologies for epigenetic research and to transfer knowledge, skills and expertise to allow the LMIC partners to independently carry out epigenetic research in future South–South collaborations. The project also allows for the development of additional hypotheses generated by the LMIC partners in the course of the research, which cannot therefore be specified here.

**Figure 1:**
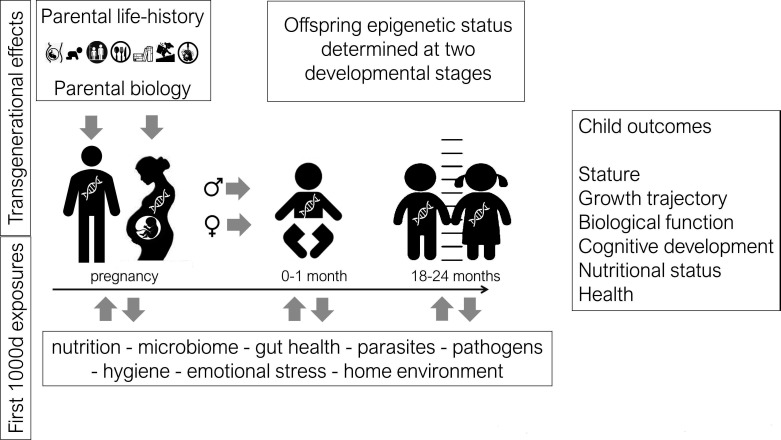
Design of observational epigenetic study.

The observational study design is essentially the same in all three countries (see Assessment of the role of gut health in childhood stunting in a multisite, longitudinal study in India, Indonesia, and Senegal: a UKRI GCRF Action Against Stunting Hub protocol. *BMJ Paeds Open, Kadia et al (in press*); Anthropometric, biochemical, dietary, morbidity and wellbeing assessments in women and children in Indonesia, India and Senegal: a UKRI GCRF Action Against Stunting Hub protocol paper. *BMJ Paeds Open, Davies-Kershaw et al (in press*)). Women are recruited (n=500 in each country, with some over-recruitment to allow for loss to follow-up) during pregnancy and the mothers and children are then followed from birth to 2 years of age (the first 1000 days). The size of the study is primarily based on the power of the epigenetic studies (see below). The overall study will assess the whole child and the environment it has been are born into. This assessment spans multiple domains including anthropometry, gut health (parasitology, enteropathy and gut microbiota composition), cognitive development (Bayley-4, INTER-NDA, OX_NDA), nutrition, food hygiene, water sanitation, food systems and home environment, and shared community values. The core epigenetics work comprises primarily laboratory genomic analysis, described below, but it also includes a Life History Questionnaire designed to inform the interpretation of the epigenetic data, particularly in relation to intergenerational effects. The design of the epigenetic work in the observational protocol, and its relationship to the other United Kingdom Research and Innovation Global Challenges Research Fund (UKRI GCRF) Action Against Stunting Hub themes, is shown in figure 1.

**Figure 2:**
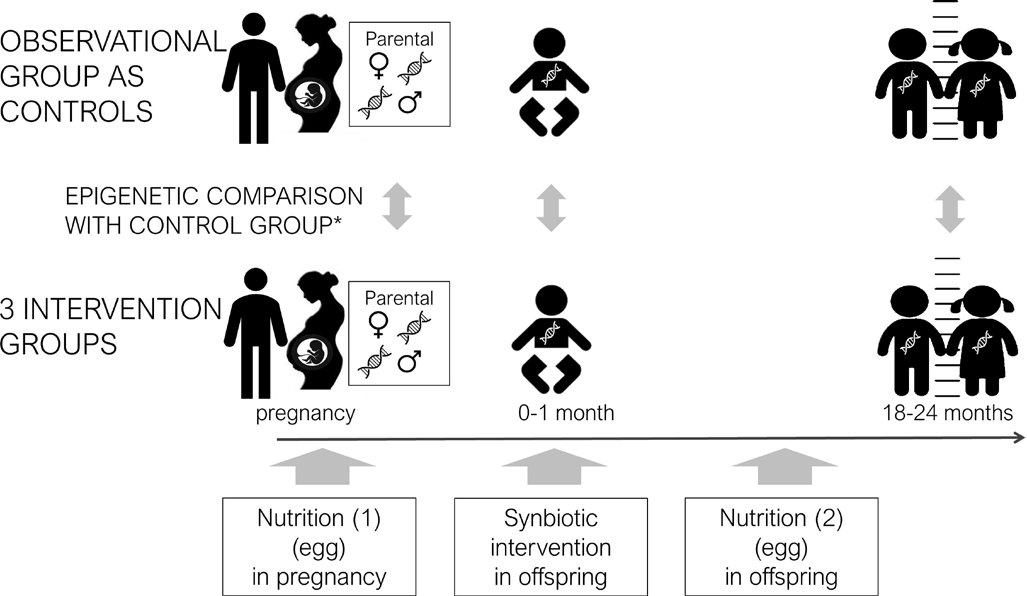
Design of intervention epigenetic study. *Some epigenetic variables are used as covariates for adjustment as relevant to intervention.

The intervention studies differ between the research centres and the primary outcomes of interest, but all will include epigenetic assessments. In Indonesia, an egg supplementation study (3 boiled eggs per week) will be carried out in n=153 women during pregnancy with n=153 controls. In India, egg supplementation (1 egg per day) will be carried out in n=350 (intervention on n=70) children (An open-label randomised controlled trial on the impact of an egg intervention on infant linear growth and cognition in Hyderabad, India: a UKRI GCRF Action Against Stunting Hub protocol paper. *BMJ Paeds Open, Kumar Banjara et al (in press*)). In Senegal, the objectives are to recruit n=236 children to receive a synbiotic with n=472 children as controls (Improving gut health and growth in early life: a protocol for an individually randomised, two-arm, open-label, controlled trial of a synbiotic in infants in Kaffrine District, Senegal. *BMJ Paeds Open, Kadia et al (in press*)). In each case, the local observational cohorts are used as controls and the epigenetic comparisons in the intervention studies are illustrated in figure 2.

In both the observational and the intervention studies, measurements of DNA methylation will be carried out in saliva samples from children collected within 1 month of birth and at 18 months of age, and from mothers and fathers around the time of birth. Anthropometry and other outcomes measures will continue to 24 months. The target is to collect the parental saliva samples within 1 month of delivery but in some cases, particularly for the fathers, this may be delayed for practical reasons and the timing of saliva sampling will be recorded. Saliva has been chosen to facilitate the easy and non-invasive collection of samples from babies and young children under potentially challenging field conditions and because saliva would be suitable for the development of epigenetic field tests of stunting risk. Samples will be collected from children in the first month postnatally using ORAcollect for Pediatrics OC-175 kits and at 18 months of age using Oragene DNA OG-675 kits. Samples will also be collected from mothers and fathers around the time of birth using Oragene DNA OG-600 kits (DNA Genotek, Ottawa, Canada). The kits include instructions for sample collection and they allow quick stabilisation of the DNA. The genomic DNA extraction will be performed using DNA Genotek Oragene prepIT.L2P reagent, according to the manufacturer’s protocol (DNA Genotek, Ottawa, Canada). For epigenetic methods requiring bisulphite conversion of DNA, the Zymo EZ DNA Methylation-Gold Bisulfite kit (Zymo Research, California, USA) will be used.

The epigenetic analysis protocol is designed to provide a general scan of the epigenome alongside high-definition sequencing. For the genome wide analysis, we will use the Illumina Infinium MethylationEPIC V.2.0 BeadChip (EPIC array), analysing around 935 000 CpG methylation sites spanning almost all known genes. Initial data processing of the raw EPIC array outputs will be performed using packages such as the Chip Analysis Methylation Pipeline^24 25^ and cell-type adjustment procedures^26^ where appropriate. The use of the EPIC array allows backward and forward comparison of the study results with a large number of epidemiological studies on methylation states relevant to child development. Statistical analysis will include epigenome-wide association in relation to selected outcomes for each work area. The EPIC array provides good representation of most coding regions but the genomic coverage is sparse and it is not able to provide information on all the epigenetic states of interest in this project. We will therefore augment this analysis with high-definition sequencing.^6^ Candidate regions are identified from our own studies and a systematic review of the literature on epigenetics, stunting and related outcomes.

Epigenetic technologies are developing rapidly and, if available before the analysis commences, the project may use improved methods as the field advances. Standardised analytical protocols have been designed to ensure consistency across the three study sites and to minimise or avoid analytical batch effects. Centrally produced DNA reference materials will be circulated to each centre to establish analytical quality before the lab analysis is undertaken. Common reference materials will also be included in the sample runs for quality assurance and quality control purposes and to ensure comparability across study sites and laboratories. Relevant quality assurance metrics will be reported in study publications.

### Statistical analysis

The statistical analysis will be carried out at the level of candidate gene/region, higher dimensional epigenetic states and epigenome-wide association, using Stata (V.17 or later), Python and R. The observational study will be analysed using applied regression models (eg, multiple linear regression, logistic regression, multilevel modelling, with adjustment for relevant covariates). The intervention studies will be analysed in terms of intention to treat and, for the nutritional interventions, the response to the actual change in nutritional status. Monte Carlo methods and simulation will be used. Dimension reduction techniques, such as principal component analysis, will be applied to high-dimensional genomic data to reduce the number of variables to those containing the essential information for use in the regression models. Epigenetic ‘type’ describes summary measures of epigenetic states in individuals derived from PCA for example. The focus of the statistical analysis is set out in box 2.

Box 2 **Focus of the epigenetic work within the UKRI GCRF Action Against Stunting Hub**

**Focus of the epigenetic work:**

- Epigenetic states characteristic of stunting and related outcomes such as cognitive development.
- Epigenetic states in non-stunted children from populations subject to stunting.
- The effect of exposures (nutrition–microbiome–gut health–parasites–pathogens–hygiene–emotional stress–home environment) in the first 1000 days of life on epigenetic states in children and risk of stunting.
- The effect of parental epigenome on offspring epigenetics and risk of stunting.
- The effect of parental life history and exposures at their birth and early childhood and at the time of the current pregnancy (season, food availability, nutritional status, physical activity) on parental epigenetic states.
- The effect of interventions (egg, synbiotic) on epigenetic state.
- The effect of baseline epigenetic type on the ability to respond to interventions.
- Comparison of the above between countries/ethnicities/populations.
- The link between epigenetics and the wider project thematic areas (nutrition–microbiome–gut health–parasites–pathogens–hygiene–emotional stress–home environment).

### Statistical power

The statistical power depends on the structure of the data (eg, covariance in methylation signatures^6^), the statistical approach and the epigenetic hypothesis. An approximate estimate can be derived for a simple comparison of epigenetic states in stunted and non-stunted children. For that comparison, the study would have a power of 80% to test 100 independent epigenetic hypotheses (p value Bonferroni adjusted for multiple testing) with 153 subjects in each group; based on a modest effect on methylation of 3% and the population standard deviation (6%) we observe using the same methodologies in other groups. For the intervention studies and subanalyses, the power may be reduced depending on the precise analysis and the number of hypotheses will be reduced accordingly. The statistical power for each analysis will be specified in publications along with the mathematical models. In addition, the data will be made available to others to allow them to generate and test their own hypotheses.

The strategy of random population sampling of n=500 is estimated to yield 150–200 stunted children, based on the estimated prevalence of stunting in the target populations (30%–40%). The national prevalence of stunting at the national level in Indonesia is 31% (Ministry of Health Republic of Indonesia. National Report of Basic Health Survey: 2018. Jakarta: National Institute of Health Research and Development; 2019 (accessed Oct 11 2021). Available from: http://labdata.litbang.kemkes.go.id/images/download/laporan/RKD/2018/Laporan_Nasional_RKD2018_FINAL.pdf) and 44% in the East Lombok District study location (Ministry of Health Republic of Indonesia. West Nusa Tenggara Province Report of Basic Health Survey: 2018. Jakarta: National Institute of Health Research and Development; 2019 https://drive.google.com/file/d/1vAcl4htxwKfKTqgRxaigCb16k3FjrnNv/view (accessed Oct 11 2021)). The prevalence of stunting in India has been on a downward trend for the past two decades but nationally in 2017, it remained high at 39% and the prevalence in the study location in Hyderabad—an urban slum—is estimated to be 61% at 18 months and 27% at 24 months of age.^27^ The rate of stunting in Senegal was approximately 26% in 2017 (Senegalese Ministry of Health National report of Continuous Demographic Health Survey (DHS-Continuous) 2017 https://www.ansd.sn (accessed 14 Feb 2022)).

### Patient and public involvement

Detailed information on participant and public involvement is presented in other protocols in this series. It involves the participation of lay members on ethical review committees and public engagement activities to publicise the research. A workstream on shared values is designed to understand the perspective of local populations affected by stunting and their views. These activities and conversations have informed the development of the research and plans for dissemination and follow-on activities.

### Dissemination

The data will be published in a range of discipline specific, cross-disciplinary, and general, peer-reviewed journals. The findings will also be translated into recommendations for policy geared towards preventing the incidence and reducing the negative impacts of childhood stunting that can be used by international organisations and governments. This will include the development of a Decision Support Tool for policy-makers based on the insights produced in the course of this work. The genomic data will be posted in public repositories such as the European Nucleotide Archive or the Gene Expression Omnibus (GEO) or equivalent repository, or as required by the relevant journal reporting the findings. Hub data will also be made available with a preference for minimal licence restrictions (typically CC-BY CC-BY-NC CC-BY-ND) as per UKRI guidance, after assessment for exploitation potential. In some cases, it may be necessary to obtain agreement on data use prior to release because of ethical or other considerations.

### Ethics

This study involves human participants. The study was approved by the relevant ethical committees in Indonesia (The Ethical Committee from Faculty of Medicine Universitas Indonesia; ND-855/UN2.F1/ETIK/PPM.00.02/2020), India (National Institute of Nutrition (ICMR), Ministry of Health and Family Welfare, Government of India, CR/04/I/2021, 01-11-2018; renewed 16-07-2021) and Senegal (The National Ethic Committee for Health Research; SN1978.31/12/2019). The study has also been approved by the Institutional Ethics Committee of the London School of Hygiene and Tropical Medicine (approval number 17915). Participants gave informed consent to participate in the study before taking part.

## Supplementary Material

Author's manuscript
